# The Efficacy of Carfilzomib Treatment in Bortezomib-Refractory Patients—Real Life Experience in a Tertiary Romanian Hospital

**DOI:** 10.3390/jcm13082171

**Published:** 2024-04-09

**Authors:** Ruxandra Irimia, Sorina Nicoleta Badelita, Sinziana Barbu, Larisa Zidaru, Ioana Loredana Carlan, Daniel Coriu

**Affiliations:** 1 Department of Hematology and Bone Marrow Transplantation, “Carol Davila” University of Medicine and Pharmacy, 050474 Bucharest, Romania; 2 Fundeni Clinical Institute, 022328 Bucharest, Romania

**Keywords:** proteasome inhibitors, bortezomib refractory, carfilzomib, efficacy, retrospective analysis, real life

## Abstract

**Background:** Proteasome inhibitors (PIs) represent one of the most effective classes of therapy for patients with multiple myeloma (MM) and are incorporated in many of the current treatment regimens. The first-generation PI, bortezomib, has shown impressive results in patients with either newly diagnosed or relapsed/refractory MM, but once patients become resistant, treatment is increasingly challenging. Although the existing data show that the second-generation PI, carfilzomib, is highly efficient, there is still limited knowledge regarding the response to carfilzomib-based therapy in bortezomib-resistant patients. The aim of this study was to evaluate carfilzomib treatment performance in bortezomib-sensitive versus -refractory patients, in a real-life eastern European country setting. **Methods:** We retrospectively evaluated 127 adult patients exposed to bortezomib with relapsed or refractory MM, that subsequently received a carfilzomib-based therapy. We investigated the differences in the overall response rate (ORR), progression-free survival (PFS), and overall survival (OS) after carfilzomib-based therapy between the two patient groups. **Results:** The ORR in the bortezomib-sensitive group was significantly higher than that in the refractory group, leading to a superior PFS in this category of patients. For patients presenting with a high cytogenetic risk, we observed a significant difference in PFS between the bortezomib-sensitive and -refractory group, while standard cytogenetic risk patients presented a similar PFS regardless of the bortezomib sensitivity status. In addition, in patients with ISS (International Staging System) stage I or II, the previous sensitivity to bortezomib correlated with an improved PFS, while for patients with ISS stage III, both groups had a comparable PFS. No significant differences in OS were observed between the two groups. **Conclusions**: In countries where novel or experimental therapies are not readily available, carfilzomib-based therapy can still be a viable therapy option for patients presenting with bortezomib-refractory status, an ISS stage III, and standard cytogenetic risk.

## 1. Introduction

Multiple myeloma (MM) is characterized by the proliferation of neoplastic plasma cells, leading to the characteristic tetrad of anemia, hypercalcemia, renal failure, and osteolytic bone lesions [1,2,3].

The primary characteristic of these malignant plasma cells is the production of excessive amounts of heavy and light monoclonal immunoglobulin chains. Consequently, drugs that target intracellular protein homeostasis are essential in combating this disease, not only by altering the clearance mechanism of excess proteins, but also by modulating the levels of key proteins involved in cell proliferation and apoptosis [4,5,6].

Proteasome inhibitors (PIs) have emerged as one of the most effective classes of therapies for patients with MM. The mechanism of action of this class of drugs involves the inhibition of the catalytically active subunits of proteasomes, which are cellular organelles responsible for the degradation of both normal and misfolded proteins. The downstream effect consists of triggering multiple pathways, including mitochondrial and endoplasmic reticulum stress, and the extrinsic death-receptor pathway that eventually leads to apoptosis [7,8,9,10].

The advent of the first-generation PI, bortezomib, represents a major breakthrough in the treatment of this condition. First approved in 2003, bortezomib revolutionized the therapy of patients with MM by increasing the 5-year survival rate from less than 30% to over 50%, and it still remains widely used in many therapy combinations among transplant-eligible and -non-eligible patients. The boronated dipeptide proteasome inhibitor reversibly hinders the 26S proteasome by binding to the chymotrypsin-like β-type 5 subunit. As a result, it induces apoptosis through several mechanisms, including the stabilization of p53, inhibition of NF-κB, suppression of Bcl-2 and STAT3, induction of endoplasmic reticulum stress, and Unfolded Protein Response [4,11,12,13,14]. Although the efficacy of bortezomib therapy is high, malignant cells eventually become resistant. Despite that the exact mechanism of resistance is not fully understood, several explanations have been postulated, including mutations in the β-type 5 subunit, the overexpression of proteasome subunits, the activation of alternative protein degradation pathways, and the upregulation of anti-apoptotic proteins [7,15,16,17,18].

Carfilzomib, a second-generation proteasome inhibitor, was approved by the FDA in 2012. It exhibits a selective and irreversible effect on the proteolytic activity of the 20S proteasome and inhibits its chymotrypsin-like activity by binding to the β-type 5 subunit [6,19,20]. Carfilzomib is highly efficient and is included in various therapeutic combinations, both in the first and subsequent relapses [6,19,21].

Treatment guidelines recommend using the CD38-targeting monoclonal antibody daratumumab rather than carfilzomib-based therapies in patients who have shown resistance to previous bortezomib-based therapies [22,23]. However, the daratumumab–bortezomib-based approach in newly diagnosed transplant-eligible (Dara-VTd/Dara-VRd) or -ineligible (Dara-Vd) patients started to become widely used, leading to a gap in the treatment options for the subsequent lines of therapy in case of treatment resistance.

Although existing data show that carfilzomib combinations are highly efficient, there is still limited knowledge regarding the response to carfilzomib-based therapy in bortezomib-resistant patients. Alsothere is a lack of evidence for the optimal usage of proteasome-based therapy in this category of patients.

## 2. Study Design

We conducted a retrospective, unicentric observational study at Fundeni Clinical Institute, the largest tertiary academic hematology center in Romania.

Using manual chart review of the electronic records, we identified 166 adult patients diagnosed with MM according to the IMWG criteria. The patients presented with relapsed or refractory disease requiring therapy and were treated with at least two cycles of carfilzomib-based combinations at our center between January 2018 and December 2022.

All patients had been previously exposed to bortezomib during the first-line therapy.

Of the total number of patients, we excluded 39 patients who either had been lost from evidence (10 patients) or had not completed at least two cycles of treatment (29 patients).

Carfilzomib has been included in the combinations Daratumumab, Carfilzomib, Dexamethasone (DKd); Carfilzomib, Lenalidomide, Dexamethasone (KRd); Carfilzomib, Dexamethasone (Kd); Carfilzomib, Pomalidomide, Dexamethasone (KPd); Carfilzomib, Cyclophosphamide, Dexamethasone (KCyD) in the standard recommended doses, according to the international and national treatment guidelines. A total of 5 patients treated before the approval of the once-weekly dose received initially for the first cycles of therapy 27 mg/m^2^ twice/week carfilzomib in combination with dexamethasone in. The rest of the patients received the standard weekly carfilzomib dose (27 mg/m^2^ for KRd and KPd, 36 mg/m^2^ for KCyd, 70 mg/m^2^ for Kd and KDd, on days 1, 8, and 15).

This study aimed to assess the overall response rate (ORR), progression-free survival (PFS), and overall survival (OS) in patients refractory to bortezomib, compared with those who maintained a response to bortezomib.

The bortezomib refractoriness was defined according to the International Myeloma Working Group (non-responsive to therapy or progressing within 60 days of the last line of therapy).

The response was evaluated according to the International Myeloma Working Group criteria. Patient follow-up was censored at the most recent hospital visit or death.

### Statistical Analysis

Patient characteristics were summarized using descriptive statistics such as mean, median, and range for quantitative variables and frequencies for qualitative variables.

Progression-free survival and overall survival were compared between the treatment groups using a log-rank test.

PFS and OS were evaluated descriptively using the Kaplan–Meier method, and the data were analyzed using Python and DATAtab analysis software.

A value <0.05 was considered statistically significant.

The overall response was compared between the groups using the Mantel–Haenszel test. Chi-square test, odds ratios (ORs) and two-sided 95% CIs, were calculated.

High cytogenetic risk was defined by the presence of del17p, t (4; 14), and t (14; 16). The Fish analysis was performed before carfilzomib therapy.

No external funding was allocated for this project.

## 3. Results

Of the 127 patients that we analyzed, 88 were sensitive to bortezomib-based therapy (group 1) and 39 were refractory (group 2).

The baseline patient characteristics in the two groups were balanced and are detailed in Table 1.

The median age was 65 years in group 1 (range, 34–84 years) and 66 years in group 2 (range, 41–78 years).

Group 1 included 43 male patients (48.86%) and 45 females (51.14%), while group 2 included 20 males (51.28%) and 19 females (48.72%).

A total of 60 patients (68.18%) in group 1 and 19 patients (48.72%) in group 2 presented with an ISS staging of I/II.

In terms of the cytogenetic risk, 37 patients (42.05%) in group 1 and 15 (38.46%) in group 2 presented with a high cytogenetic risk.

The median time from diagnosis was 6.14 years for group 1 and 5.78 years in group 2.

The patients were exposed to a median of two previous lines of therapy in both groups: group 1 (1–5) and group 2 (1–6).

Regarding the carfilzomib therapeutic combinations, KRd was the most frequently used combination in 59 patients (67.04%) in group 1 and 20 patients in group 2 (51.28%).

A total of 19 patients (21.59%) in group 1 and 14 (35.89%) in group 2 were treated with the Kd combination. Seven patients (7.95%) and three patients (7.69%) were exposed to the DKd combination in group 1 and group 2, respectively.

The other combinations recorded were KCyD (three patients, 3.41%, and one patient, 2.56%) and KPd (zero patients, 0%, and one patient, 2.56%).

The reasons for treatment discontinuation are summarized in Table 2. A total of 52 patients (59.09%) in group 1 and 30 patients (76.92%) in group 2 discontinued treatment due to disease progression (*p* = 0.53). In total, 21 patients (23.86%) in group 1 and 5 patients (12.82%) in group 2 completed the 18 cycles of the fixed-duration KRd combination. Adverse events related to therapy resulted in treatment discontinuation for a minority of patients, six patients (6.82%) in group 1 and one patient (2.56%) in group 2 (*p* = 0.333). These included grade 4 and 5 infectious events, drug-induced hepatotoxicity, and cardiovascular events. None of the patients discontinued treatment due to hematologic adverse events. Concerning the cardiovascular adverse events, one patient in group 1 developed grade 3 myocardial infarction, and one patient in each group developed persistent grade ≥3 hypertension and required permanent treatment discontinuation. Notably, one patient developed carfilzomib-induced atypical hemolytic uremic syndrome during the first cycle of therapy; however, this case was not included in the final analysis since it did not fulfill the inclusion criteria (the completion of a minimum of two complete cycles of therapy).

**Table 1:** Patient characteristics.

	Group 1—Bortezomib-Sensitive (*n* = 88) Median (Range)	Group 2—Bortezomib-Refractory (*n* = 39) Median (Range)
**Age** (years)	65 (34–84)	66 (41–78)
**Sex**		
Male	43 (48.86%)	20 (51.28%)
Female	45 (51.14%)	19 (48.72%)
**Race**		
White	100%	100%
Black	0%	0%
Asian	0%	0%
Multiple	0%	0%
**ISS stage**		
I–II	60 (68.18%)	19 (48.72%)
III	28 (31.82%)	20 (51.28%)
**Cytogenetic risk**		
Standard	22 (25.00%)	13 (33.33%)
High	37 (42.05%)	15 (38.46%)
Not available	29 (32.95%)	11 (28.20%)
**Number of previous lines of therapy** Median (range)	2.5 (1–5)	2.6 (1–6)
**Time from diagnosis (years)** (median)	6.145	5.78
**Carfilzomib combination**		
KRd	59 (67.04%)	20 (51.28%)
Kd	19 (21.59%)	14 (35.90%)
DKd	7 (7.96%)	3 (7.69%)
KCyD	3 (3.41%)	1 (2.56%)
KPd	0 (0%)	1 (2.56%)

Abbreviations: KRd (Carfilzomib, Lenalidomide, Dexamethasone), Kd (Carfilzomib, Dexamethasone), DKd (Daratumumab, Carfilzomib, Dexamethasone), KCyD (Carfilzomib, Cyclophosphamide, Dexamethasone), KPd (Carfilzomib, Pomalidomide, Dexamethasone).

**Table 2:** Causes of treatment discontinuation.

Reason for Treatment Discontinuation	Group 1—Bortezomib-Sensitive (*n* = 88)	Group 2—Bortezomib-Refractory (*n* = 39)
**Disease progression**	52 (59.09%)	30 (76.92%)
**Therapy completion**	21 (23.86%)	5 (12.82%)
**Ongoing therapy**	9 (10.23%)	3 (0.77%)
**Discontinuation due to adverse events**		
Grade ≥4 infectious events	3 (0.34%)	0 (0.00%)
Grade ≥3 cardiovascular events	2 (2.27%)	1 (2.56%)
Drug-induced hepatotoxicity	1 (1.14%)	0 (0.00%)

The proportion of patients achieving at least a PR was statistically significantly higher in the bortezomib-sensitive group (84, 95.5%), compared to the bortezomib-refractory group (32, 82.05%) (odds ratio (OR), 4.593 [95% CI, 1.259, 16.758], *p* = 0.032).

The overall response rates are detailed in Table 3.

**Table 3:** Treatment response.

Response	Group 1—Bortezomib-Sensitive (*n* = 88)	Group 2—Bortezomib-Refractory (*n* = 39)
**ORR**	84 (95.46%)	32 (82.05%)
CR	25 (28.41%)	1 (2.56%)
VGPR	50 (56.82%)	21 (53.85%)
PR	9 (10.28%)	10 (25.64%)
**SD**	1 (1.14%)	3 (7.69%)
**PD**	3 (3.41%)	4 (10.26%)

ORR (Overall Response Rate), CR (Complete Response), VGPR (Very Good Partial Response), PR (Partial Response), SD (Stable Disease), PD (Progressive Disease).

The median follow-up for PFS was 310 days.

**Figure 1:**
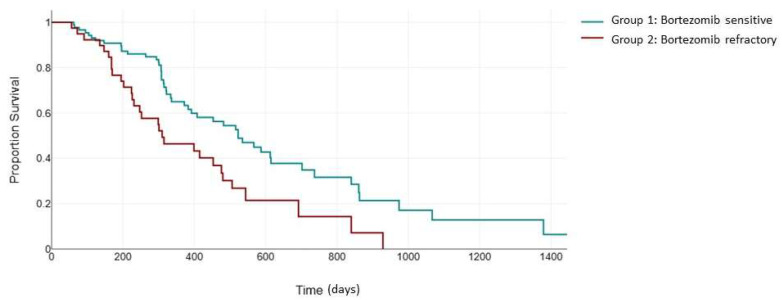
Median PFS (days) between the bortezomib-sensitive group (green) and the bortezomib-refractory group (red).

As expected, the superior response rates in group 1 translated into an improved PFS. The median PFS in the bortezomib-sensitive group was 523 days, which was significantly higher than the median PFS of 310 days in the bortezomib-refractory group (*p* = 0.003) (Figure 1).

**Figure 2:**
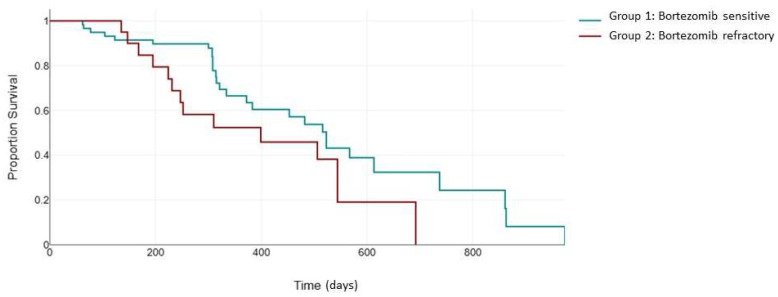
Median PFS (days) between the bortezomib-sensitive group (green) and the bortezomib-refractory group (red) in patients treated with the KRd combination.

Next, we evaluated PFS in the 79 patients uniformly treated with the KRd combination (Figure 2). The ASPIRE trial validated the efficacy of the KRd combination in patients receiving one to three previous lines of therapy, with a median PFS of 26.3 months in the KRd arm [24]. The inclusion of patients who were previously exposed to bortezomib was permitted if they showed no disease progression during therapy [24]. The median PFS for KRd vs. Rd was 24.4 versus 16.6 months for patients with previous bortezomib exposure and 30.3 versus 18.2 months for patients without previous bortezomib exposure. In our study, the median PFS in the bortezomib-sensitive group was 523 days (95% CI: 372 to 613) compared to 399 days (95% CI: 231 to 506) in the bortezomib-refractory group. Although the difference in median PFS did not reach statistical significance (*p* = 0.107), probably due to the small sample size, the Kaplan–Meier curves clearly demonstrates a notable separation, suggesting a potential clinical relevance of the observed trend, warranting further exploration and consideration in the context of clinical implications.

We next evaluated the PFS in response to carfilzomib therapy in the high-cytogenetic-risk patients. In the ENDEAVOR clinical trial (Kd vs. Vd), carfilzomib showed a clear advantage in terms of PFS and OS in patients with high-risk cytogenetics compared to the first-generation PI, bortezomib [25].

**Figure 3:**
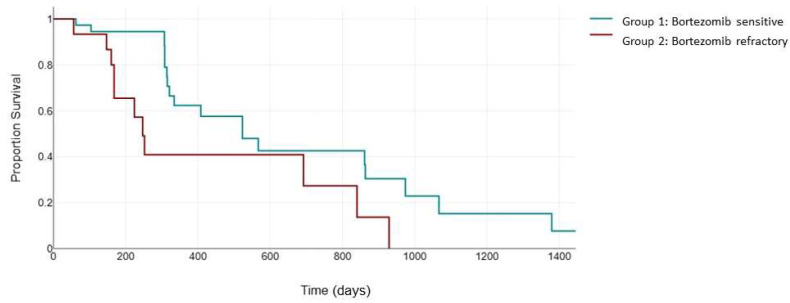
Median PFS (days) between the bortezomib-sensitive group (green) and the bortezomib-refractory group (red) in the high-cytogenetic-risk patients.

The KRd combination also improved the negative impact of del17p and t (4;14) in the ASPIRE clinical trial (23.1 months versus 13.9 moonths, HR: 0.703 [0.426, 1.160], *p* = 0.0829) [24]. However, it is still unclear how bortezomib sensitivity status interferes with the efficacy of carfilzomib in this category of patients. In our study, for the patients with high-cytogenetic-risk features, the median PFS was 523 days (95% CI, 321 to 863) in group 1, statistically significantly higher than that in group 2, 247 days (95% CI, 168 to 692), with a *p* = 0.012 (Figure 3).

**Figure 4:**
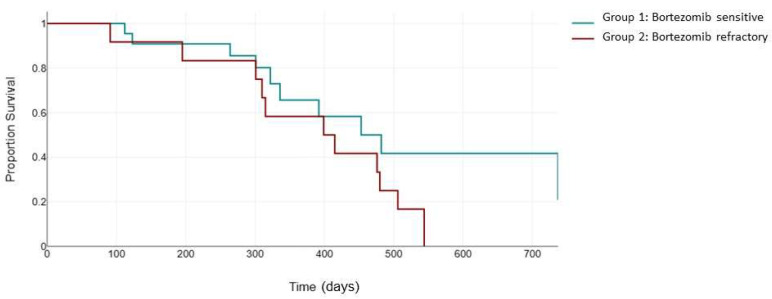
Median PFS (days) between the bortezomib-sensitive group (green) and the bortezomib-refractory group (red) in the standard-cytogenetic-risk patients.

However, when evaluating the standard cytogenetic risk population, there was no significant difference in PFS between the two groups (482 days vs. 399 days, *p* = 0.125) (Figure 4).

Next, we evaluated the impact of the number of previous lines of therapy on PFS. The ENDEAVOR clinical trial showed that the patients receiving the Kd combination with a previous exposure to only one prior line of therapy showed a longer PFS compared to the patients that were exposed to two or three previous lines of therapy (22.2 versus 14.9 months, respectively) [25]. In the ASPIRE trial, the PFS for the KRd group was 29.6 months for patients exposed to only one line of therapy, compared to 25.8 months in patients with two or more previous lines [24]. Due to the relatively small sample size, in our study, we only evaluated the PFS differences between the two groups for patients that received ≥2 lines of therapy.

**Figure 5:**
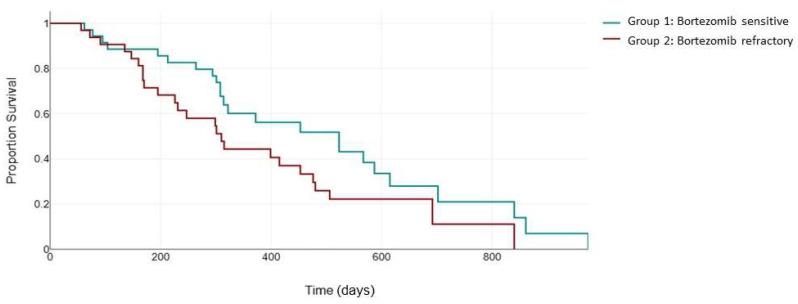
Median PFS (days) between the bortezomib-sensitive group (green) and the bortezomib-refractory group (red) in patients that received 1 or 2 previous lines of therapy.

For these patients, the median PFS was 523 days for group 1 (95% CI, 314 to 587) compared to 310 days for group 2 (95% CI, 226 to 453). Although this difference was not statistically significant (*p* = 0.064), the trend tended to favor group 1 (Figure 5).

Another important aspect that we analyzed is the efficacy of carfilzomib-based therapies in the two groups of patients based on previous exposure to immunomodulatory drugs. In the ASPIRE trial, previous exposure to lenalidomide, and to a lesser extent thalidomide, was associated with a lower PFS in the KRd arm [24]. The median PFS for KRd was 29.6 months in the patients without previous thalidomide exposure versus 25.9 months with previous thalidomide exposure. Lenalidomide exposure resulted in a 19.4-month PFS for KRd compared to a 28.7-month PFS in the absence of lenalidomide exposure. This effect has also been observed in the ENDEAVOR trial, where prior lenalidomide exposure decreased the PFS from 17.6 months in the ITT population to 12.9 months for the Kd arm [25].

In our cohort, we observed no statistically significant difference in PFS between the two groups, regardless of immunomodulatory drug exposure status. In patients with previous immunomodulatory drug exposure, the median PFS was 322 days in group 1 versus 301 days in group 2 (*p* = 0.109). In the absence of IMiD exposure, the median PFS was 535 days in group 1 versus 544 days in group 2 (*p* = 0.446).

Another factor associated with response to treatment included in our analysis was ISS staging. In both ASPIRE and ENDEAVOR clinical trials, the carfilzomib arm showed a superior PFS compared to the control arm regardless of ISS staging [24,25]. However, it is unclear how the efficacy of carfilzomib is influenced by ISS staging, depending on previous sensitivity status to bortezomib.

**Figure 6:**
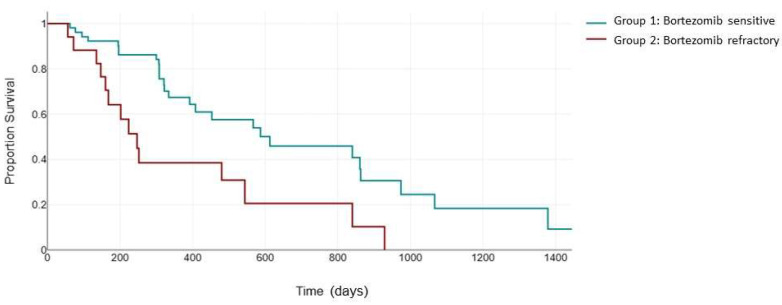
Median PFS (days) between the bortezomib-sensitive group (green) and the bortezomib-refractory group (red) in patients with an ISS stage of I or II.

In our cohort, for an ISS stage of I or II, patients in group 1 achieved a significantly higher median PFS compared to group 2 when treated with a carfilzomib-based combination, 613 days (95% CI, 392 to 861) compared to 247 days (95% CI, 160 to 480) (*p* = 0.002) (Figure 6).

**Figure 7:**
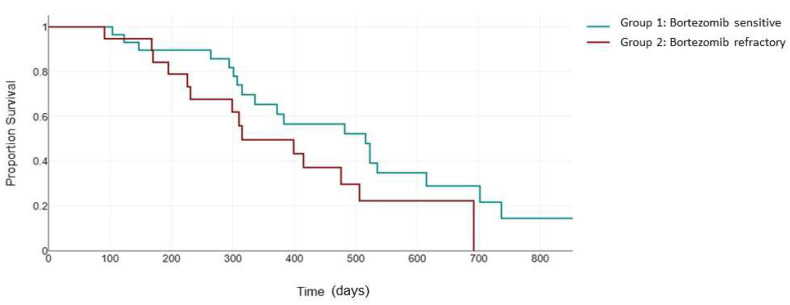
Median PFS (days) between the bortezomib-sensitive group (green) and the bortezomib-refractory group (red) in patients with an ISS stage of III.

Interestingly, for patients with an ISS of III, we observed no statistically significant difference in PFS between the two groups (516 days versus 315 days, *p* = 0.114) (Figure 7).

Along with PFS, the prolongation of OS is another important endpoint in the treatment of patients with MM. The improved OS in this incurable disease, led by the emergence of novel therapies, has also made the management of therapy in the relapsed/refractory setting more challenging. The ENDEAVOR clinical trial is considered one of the first trials to present substantial evidence of extended OS for a novel therapy compared to the standard of care. In this setting, the Kd arm showed a 47.6 median OS compared with 40 months in the Vd arm (*p* = 0.010) [25]. However, subsequent data suggest that previous exposure to another PI might be associated with a decrease in OS.

In the ASPIRE study, previous bortezomib exposure was associated with an OS of 45.9 months in the KRd arm compared with NE in the overall treated population [24].

In the CANDOR trial, in patients that were previously refractory to Pis compared to non-refractory, in the DKd arm, the OS was 43.2 months compared to NE, while for the Kd arm, the OS was 30.0 months compared to 51.8 months [26].

**Figure 8:**
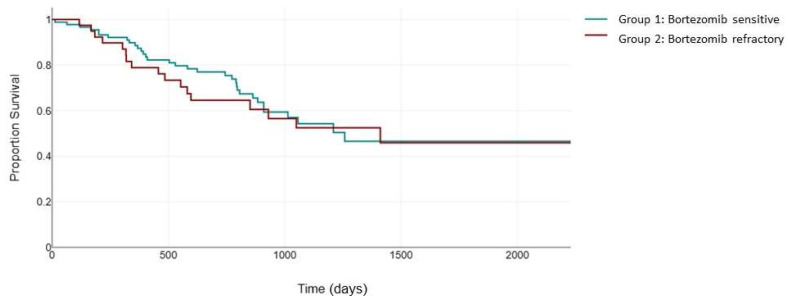
Median OS (days) between the bortezomib-sensitive group (green) and the bortezomib-refractory group (red).

In our study, overall, the OS in the bortezomib-sensitive group was 1258 days, which was not statistically different from the OS in the bortezomib-refractory group (1411 days, *p* = 0.632) (Figure 8).

**Figure 9:**
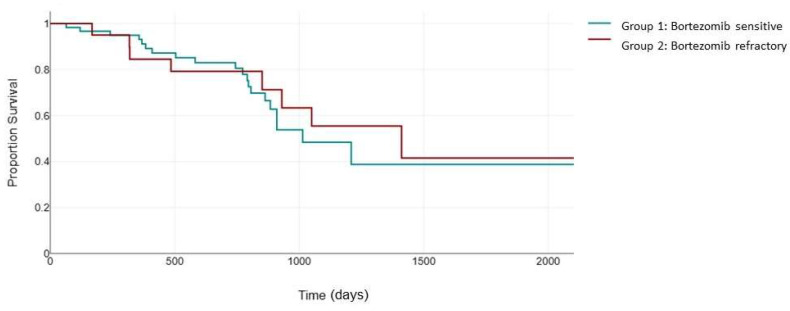
Median OS (days) between the bortezomib-sensitive group (green) and the bortezomib-refractory group (red) in the KRd-treated patients.

When considering the uniformly treated KRd population, the OS did not differ significantly between the two groups, probably due to the small sample size. The median OS was 1014 days for group 1, compared to 1411 days for group 2, *p* = 0.629 (Figure 9).

For patients exhibiting a high cytogenetic risk, the OS did not differ between the KRd arm and the control arm in the ASPIRE study (36.0 months versus 36.0 months) [24]. In the CANDOR study, the OS for the DKd arm was 34.3 months for the high-risk subgroup compared to NE in the standard-risk patients [26]. For the control (Kd), the OS was 17.1 months versus 38.2 months in the high-risk- versus standard-cytogenetic-risk patients, respectively.

In our study, when considering cytogenetic risk, it seems that high cytogenetic risk does not play a role in the OS between the two groups.

**Figure 10:**
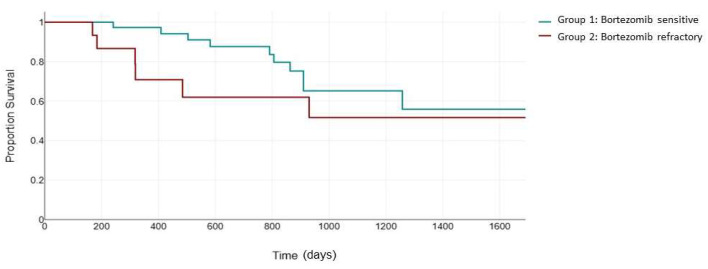
Median OS (days) between the bortezomib-sensitive group (green) and the bortezomib-refractory group (red) in the high-cytogenetic-risk patients.

In the high-cytogenetic-risk group, although there was a clear trend favoring group 1, the OS difference was not statistically significant—1068 days for group 1, compared to 693 days in group 2 (*p* = 0.246) (Figure 10).

When considering the number of previous lines of therapy, the ASPIRE study showed an OS of 47.3 months for patients who received one previous line of therapy in the KRd arm compared to 48.8 months for patients who received two or more previous lines of therapy [24].

**Figure 11:**
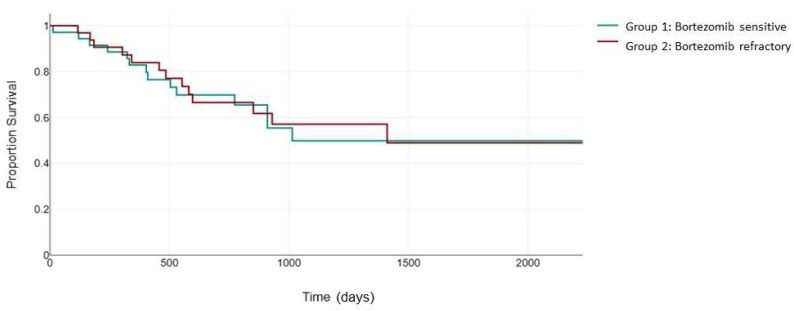
Median OS (days) between the bortezomib-sensitive group (green) and the bortezomib-refractory group (red) in patients with 2 or more prior lines of therapy.

In the CANDOR study, for patients with one prior line of therapy versus two or more prior lines of therapy, the OS was NE versus 45.2 in the DKd arm compared to 51.8 months versus 35.4 months in the Kd arm [26]. In our study, the patients that received ≥2 lines of therapy showed no significant difference between the two groups in terms of OS (1014 days compared to 1411 days, *p* = 0.814) (Figure 11).

## 4. Discussion

Carfilzomib is highly efficient in the treatment of relapsed MM; however, there is still a gap in knowledge regarding its efficacy in patients refractory to previous bortezomib treatment.

The current ESMO guidelines recommend the following carfilzomib combinations for patients with R/R MM: DKd, KRd, IsaKd, Kd [22]. However, in the bortezomib-resistant population, the treatment choices become restricted. Second-line options are limited to DaraKd and IsaKd, while subsequent lines of therapy often involve pomalidomide-based combinations. Moreover, since daratumumab has become extensively used in the first line of treatment in combination with ImiDs and/or bortezomib, selecting a subsequent line of therapy is becoming increasingly challenging. This is particularly relevant in low-to-middle-income countries, where alternative therapeutic options are scarce.

Existing clinical trials have proven the efficacy of carfilzomib combinations in patients with relapsed MM; however, in many cases, bortezomib-refractory patients have been excluded from enrollment.

The CANDOR clinical trial, comparing the efficacy of Daratumumab–Kd to Kd, was one of the few trials that included patients refractory to previous PI therapy. The study reports that this category of patients had a more dismal prognosis compared to the overall study population [26].

In addition, in the ARROW clinical trial, patients who were refractory to bortezomib as their last line of therapy had an ORR of 18.6%, compared to 28.3% for patients who did not receive bortezomib as their most recent line of therapy [27].

This study aimed to retrospectively evaluate the efficacy of carfilzomib-based combinations in patients with relapsed or refractory multiple myeloma. Previous bortezomib-refractory status is associated with significantly lower response rates than their bortezomib-sensitive counterparts, leading to a statistically lower PFS.

We also observed a strong correlation between the ISS stage and the carfilzomib therapy response between the two groups. Patients in group 1 exhibiting an ISS of I or II presented with improved PFS when exposed to carfilzomib compared to patients in group 2. However, for patients with an advanced disease and an ISS of III, there was no significant difference in the PFS between the two groups. The cytogenetic risk also represents an important decision factor when selecting therapy. Intriguingly, for patients with a standard cytogenetic risk treated with carfilzomib, we observed no PFS difference, regardless of their previous bortezomib sensitivity status. This suggests that carfilzomib may be a viable treatment option for standard-risk patients irrespective of their previous bortezomib response. However, for patients with high cytogenetic risk, the performance of carfilzomib-based therapy in group 2 was suboptimal, indicating that alternative treatment options, including clinical trial enrollment, should be considered in this population. In addition, we did not detect any significant difference in PFS between the two patient groups when considering the number of previous lines of therapy (≥2). However, the survival analysis tended to favor the use of carfilzomib in group 1.

These data suggest that carfilzomib-based therapy is a viable treatment option for patients with ISS stage III and standard cytogenetic status, regardless of prior bortezomib treatment.

The existing data we collected did not offer sufficient statistical power to detect significant changes in the OS between the two groups, although for the standard-risk population, the OS was almost double in the bortezomib-sensitive patients compared to the bortezomib-refractory group.

The limitations of this study include its retrospective design and lack of uniformity in the therapeutic combinations used to treat the patient population, which may have introduced potential biases and limitations in data interpretation and outcomes.

Despite collecting a robust number of patients, this study’s statistical power was not sufficient to detect differences between the traditionally described patient subgroups.

## Data Availability

The data presented in this study are available on request from the corresponding author.

## Abbreviations

KRd (Carfilzomib, Lenalidomide, Dexamethasone), Kd (Carfilzomib, Dexamethasone), DKd (Daratumumab, Carfilzomib, Dexamethasone), KCyD (Carfilzomib, Cyclophosphamide, Dexamethasone), KPd (Carfilzomib, Pomalidomide, Dexamethasone), IsaKd (Isatuximab, Carfilzomib, Dexamethasone), IMiDs (immunomodulatory drugs), PIs (Proteasome Inhibitors), ORR (Overall Response Rate), CR (Complete Response), VGPR (Very Good Partial Response), PR (Partial Response), SD (Stable Disease), PD (Progressive Disease).
